# Medication-Related Problems in Liver Transplant Recipients in the Outpatient Setting: A Dutch Cohort Study

**DOI:** 10.3389/fphar.2021.637090

**Published:** 2021-04-27

**Authors:** Midas B. Mulder, Sander D. Borgsteede, Sarwa Darwish Murad, Catelijne S. Landman, Herold J. Metselaar, Nicole G.M. Hunfeld

**Affiliations:** ^1^ Department of Hospital Pharmacy, Erasmus MC, University Medical Center Rotterdam, Rotterdam, Netherlands; ^2^ The Erasmus MC Transplantation Institute, Erasmus MC, University Medical Center Rotterdam, Rotterdam, Netherlands; ^3^ Department of Clinical Decision Support, Health Base Foundation, Houten, Netherlands; ^4^ Department of Hepatology, Erasmus MC, University Medical Center Rotterdam, Rotterdam, Netherlands; ^5^ Department of Intensive Care, Erasmus MC, University Medical Center Rotterdam, Rotterdam, Netherlands

**Keywords:** medication-related problems, hospital pharmacy, liver transplantation, medication safety, medication review

## Abstract

**Background:** After liver transplantation (LTx), adherence to immunosuppressive medication and avoidance of contra-indicated drugs is essential for long-term survival. This study aimed to investigate the prevalence, types and severity of medication-related problems (MRPs) and interventions initiated by a clinical pharmacist (CP) in a cohort of LTx recipients in the outpatient setting.

**Method:** This study was a retrospective, observational study in LTx recipients that visited the outpatient clinic for an annual check-up. A 20-minutes consultation with a CP consisted of medication reconciliation and consultation about medication, adherence, and adverse drug reactions (ADRs). Discrepancies between actual and intended drug use, and MRPs were identified and the severity of MRPs was assessed. Potential interventions were discussed with the patient and the treating physician and evaluated after one year.

**Results:** The CP counseled 64 LTx recipients and found 96 discrepancies in 37 patients. Most discrepancies (60.4%, *n* = 58) concerned missing medications.

In total, 98 MRPs were identified in 53 patients (median 2; range 1-5 per patient), with a total of 113 interventions. Most frequent MRPs were: ADRs (22.4%, *n* = 22), nonadherence (19.3%, *n* = 19), unnecessary drugs (16.3%, *n* = 16) and undertreatment (12.2%, *n* = 12). Interventions most frequently proposed included optimization of dosage regimen (21.2%, *n* = 24), individualized recommendation regarding compliance (16.8%, *n* = 19) and drug discontinuation (12.4%, *n* = 14). After one year, 15 of the 19 patients (79%) experienced no longer compliance issues and 27 of the 29 patients (93%) used no drugs with indication issues anymore.

**Conclusion:** The CP in an outpatient monitoring program for LTx recipients can signal relevant discrepancies and MRPs. This leads to interventions that are accepted by both the patients and the physicians, with a positive effect after one year.

## Introduction

Liver transplantation (LTx) is the preferred treatment in patients with end-stage liver disease and hepatocellular carcinoma with 1-year patient survival exceeding 80%. However, survival rates gradually decline over time with 5-year and 10-year patient survival rates of respectively 71 and 61 % (European Liver and Intestine Transplant Association, 1985). Adherence to immunosuppressive medication and avoidance of contra-indicated drugs are two potential modifiable risk factors to improve long-term outcome (Neuberger et al., 2017). In addition, due to the development of comorbidities, LTx recipients will usually end up with multiple drugs over the years. Over 30 years of experience, we learned that medication errors contribute to a substantial amount of unplanned hospitalizations (Brennan et al., 1991; Beijer and de Blaey, 2002). In the Netherlands, the Hospital Admissions Related to Medication (HARM) study showed that 5.6% of all unplanned hospitalizations are drug related and that 46% of these were potentially preventable (Leendertse et al., 2008). Therefore, identification and management of medication-related problems (MRPs) opens opportunities to improve medication safety. Several studies have shown that a medication review might contribute to the detection, prevention and management of MRPs in all sorts of settings (Vink et al., 2011; Roane et al., 2014).

MRPs are defined as events or circumstances involving drug therapy that actually or potentially interferes with desired health outcomes (Pharmaceutical Care Network Europe (PCNE), 2019). Examples of MRPs are adverse drug reactions, drug interactions, nonadherence, unnecessary drug use and untreated indications.

In North-America clinical pharmacists (CP) have been involved in the direct patient care in transplantation since the early 1970s. (Sam et al., 2018). In the Netherlands, pharmacists working in the hospital as CPs are more recently starting to be involved in the direct care for hospitalized patients (Bosma et al., 2008). Only a few CPs are involved in the out-patient care as well. As far as we know, no CP has been structurally involved in the out-patient care of liver transplant recipients in the Netherlands.

Taber et al. showed that MRPs and adverse drug events commonly occur in kidney transplant recipients resulting in higher rates of acute rejection and lower graft survival rates (Taber et al., 2012). Despite the fact that LTx recipients take comparable drugs as kidney transplant recipients, so far no study describes the prevalence and types of MRPs in LTx recipients and the impact of interventions initiated by a CP in this population. By investigating MRPs in LTx recipients more information about MRPs in the transplantation population becomes available resulting in more awareness, possibly earlier detection of MRPs and better prevention strategies.

This study aimed to investigate the prevalence and types of MRPs in a cohort of liver transplant recipients in the outpatient setting in one of the three liver transplant centers of the Netherlands. The secondary objectives were to investigate the severity of the MRPs and the type and impact of interventions initiated by a CP to improve medication use.

## Method

### Ethics Approval

This study was a retrospective, observational study conducted between September–December 2018 at the Erasmus MC University Medical Center Rotterdam, the Netherlands and was approved by the Medical Ethics Committee of the Erasmus University Medical Center (MEC-2019–0784).

### Study Design

Since 1986, 1271 liver transplantations have been performed in 1116 adult patients at the Erasmus University Medical Center, Rotterdam, the Netherlands. Currently, 713 liver transplantation (LTx) recipients are still alive and 671 are seen at least annually at the outpatient clinic. The other 42 recipients are lost to follow-up. Adult LTx recipients were eligible if they were scheduled for an annual, multidisciplinary medical check-up at the outpatient clinic. During this annual medical check-up, the recipient is seen by a hepatologist or specialized nurse practitioner and a social worker. Since hospital pharmacists have knowledge and experience regarding the pharmacotherapy and comorbidities of LTx recipients, a CP was added to the LTx program of the Erasmus University Medical Center in September 2018 as part of integrated patient care. A newly established 20-minute face-to-face consultation with the CP was added to the annual check-up. Patients were asked to bring their own medication and a list of prescriptions as registered by their community pharmacy. The consultation consisted of medication reconciliation and a conversation about medication, adherence, adverse drug reactions (ADRs) and drug use. Potential interventions were discussed with the patient and the hepatologist and after consensus initiated by the CP. In total, the CP spent approximately 45°minutes per patient for the preparation of the consultation, the consultation with the patient and the evaluation afterwards with the LTx team. All findings were registered in the patient’s electronic medical records for further follow-up by the hepatologist or the CP. The findings of the annual check-up were reported to the primary care physician by the hepatology department. The consulting pharmacist, MBM, completed a special training on the treatment of LTx patient’s through courses and a mentorship with a transplant hepatologist. One year after the first consultation, all MRPs and proposed interventions per patient were evaluated by the CP in the annual check-up in order to evaluate the clinical impact of the outpatient monitoring program.

### Patients

**FIGURE 1:**
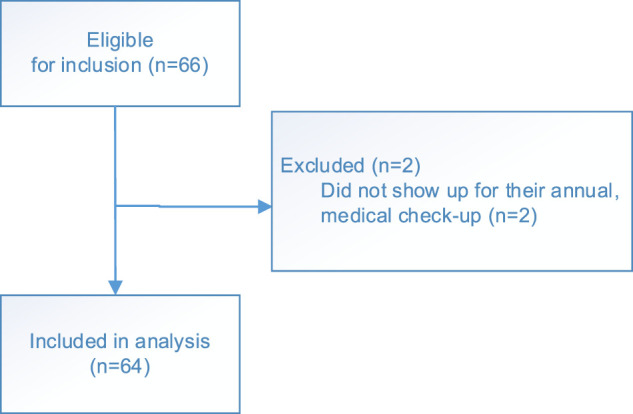
Flow chart.

Adult LTx recipients scheduled for an annual, multidisciplinary medical check-up at the outpatient clinic between September–December 2018 were included in this analysis (Figure 1).

### Data Collection

Socio-demographic and clinical parameters were extracted from patient’s electronic medical records in the hospital information system. The following information was collected from patient’s electronic medical records: gender, age, presence of comorbidities, reason for and date of the transplantation, information about re-transplantation, medication according to the patient’s electronic medical records in the hospital and according to the list of prescriptions distributed by their community pharmacy.

During the face-to-face consultation, the CP retrieved information about drug use reported by the patient, adherence, adverse drug reactions, untreated conditions, problems with medication use and proposed interventions. All information was registered in a data extraction file. Next, MRPs were identified by reviewing all information documented by the CP. No additional information from the patient’s electronic medical record was necessary for the assessment of the MRPs.

### Assessment of Medication-Related Problems

The registered information was categorized into predefined categories of MRPs. These categories were based on the classification of the Pharmaceutical Care Network Europe (PCNE) Classification V 9.0. and the classification used by Hayward et al. that was applied in patients with cirrhosis (Pharmaceutical Care Network Europe (PCNE), 2019; Hayward et al., 2019).

Each identified MRP was categorized and for one MRP, several interventions could have been proposed. All MRPs and interventions were independently categorized by two pharmacists (MBM and SDB). Next, they compared their classifications and when dissensus existed, the panel members reviewed their own classifications and discussed these until consensus was reached.

### Assessment of the Severity of the Medication-Related Problems

The severity of the MRPs was assessed with the National Coordinating Council for Medication Error Reporting and Prevention (NCC MERP) index (Hartwig et al., 1991). This classification is widely used and categorizes medication errors (for example MRPs) into nine categories (A–I) based on the severity of the related patient outcomes. The first categories (A–D) are associated with errors that caused no or potential harm to the patient. Categories (E–I) are associated with errors that caused harm or even death to the patient.

Each identified MRP was categorized according to the NCC MERP index. The rating was based on the potential impact of the detected MRP on the patient’s health status. Several common MRPs were rated in a standardized way (European Liver and Intestine Transplant Association, 1985): nonadherence (Neuberger et al., 2017), experience complexity in dosage regimen and (Brennan et al., 1991) ADRs in category D (error caused potential harm to patients) (Beijer and de Blaey, 2002); use of anticoagulants without indication and (Leendertse et al., 2008) use of contraindicated drugs in category E (error caused temporary harm and required intervention). All MRPs were independently categorized by two pharmacists (MBM and NH). Next, they compared their classifications and when dissensus existed, the panel members reviewed their own classifications and discussed these until consensus was reached.

### Statistical Analysis

No formal sample size calculation was performed. We included all patients in the analysis who received a consultation with the CP during their annual, multidisciplinary medical check-up at the outpatient clinic.

Variables were described with descriptive statistics: n (%) for nominal and ordinal variables and median (inter‐quartile range, IQR) for the continuous variables. Statistical software, SPSS for Windows, version 24 (SPSS, Chicago, IL), was used for the analysis. No statistical tests were performed.

## Results

The CP counseled 64 patients with a median age of 59.5°years (IQR: 47–66) and a median of seven medications (IQR: 5–8). The most prevalent indication for LTx was cirrhosis. Frequent comorbidities were chronic kidney disease (*n* = 26), cardiovascular disease (*n* = 26), and diabetes mellitus (*n* = 19); 11 patients had no comorbidities. Table 1 presents the clinical and demographical characteristics of the cohort.

**TABLE 1:** Clinical and demographical characteristics.

		Patients (*n* = 64)
Age (year) (median, IQR)		59.5 (47–66)
Gender	Male (n, %)	37 (57.8%)
Indication liver transplantation	Cirrhosis^a^	30 (46.9%)
	Hepatitis B Virus	11 (17.2%)
	Hepatocellular carcinoma	9 (14.1%)
	Acute Liver Failure	7 (10.9%)
	Hepatitis C Virus	7 (10.9%)
	Other^b^	11 (17.2%)
Time after transplantation (years) (median, IQR)		8 (3.5–12.5)
Re-transplantation	No	59 (92.2%)
	Yes	5 (7.8%)
Presence of a comorbidity^c^	Cardiovascular Disease	26 (40.6%)
	Chronic Kidney Disease^d^	26 (40.6%)
	Diabetes Mellitus	19 (29.7%)
	None	11 (17.2%)
	Gastrointestinal	8 (12.5%)
	Other^e^	17 (26.6%)
Number of drugs on medication list during consultation (median, IQR)		7 (5–8)

IQR, inter-quartile range.

a Cirrhosis was caused by Primary Sclerosing Cholangitis (*n* = 17), alcohol abuse (*n* = 3), nonalcoholic steatohepatitis (*n* = 2), Primary Biliary Cholangitis (*n* = 1) and cryptogenic cirrhosis (*n* = 7).

b Other includes: Autosomal Dominant Polycystic Kidney disease (*n* = 3), Alpha-1 Antitrypsin Deficiency (*n* = 2), Hemochromatosis (*n* = 2), Hepatitis D Virus, Budd Chiari, hepatopulmonary syndrome, Wilson’s disease.

c Comorbidity: Every comorbidity is counted separately.

d Chronic Kidney disease is defined according to the KDIGO guidelines (Levey et al., 2005).

e Other includes: neurological (*n* = 5), hematological (*n* = 3), dermatological (*n* = 2), thyroid disorders (*n* = 2), psychiatric (*n* = 2), immunological (*n* = 1), pulmonary (*n* = 1) and rheumatological comorbidities (*n* = 1).

### Medication Discrepancies During Consultation

Table 2 presents an overview of the medication discrepancies during consultation. In 37 patients (57.8%), one or more discrepancies were found in the medications registered in the hospital information system and the ones actually used by the patient. Most discrepancies (60.4%) involved missing medications (i.e. medications used by the patient but not registered in the chart). For example, medicines prescribed by the general practitioner as inhaled medication, antihypertensive agents or oral anti-diabetics. All discrepancies in the patient’s electronic medical records in the hospital were subsequently corrected by the hepatologist treating the patient. In 27 patients (42.2%) no discrepancy was found.

**TABLE 2:** Discrepancies between medication recorded in the patient’s electronic medical records and actual medication used by patient.

Type of discrepancy	Number of discrepancies (*n* = 96)	Number of patients with ≥1 discrepancy (*n* = 64)	Example of discrepancies
Missing medication in patient’s electronic medical records (n, %)	58 (60.4 %)	27 (42.2 %)	Tiotropium inhaler 18 ug was initiated by the general practitioner and not registered in the patient’s electronic medical record
Unnecessary medication in patient’s electronic medical records (n, %)	23 (24.0 %)	14 (21.9 %)	Hydrochlorothiazide tablets or iron tablets were registered as active medication in the patient’s electronic medical record whereas another physician advised the patient to stop the tablets
Incorrect dose or dose frequency in patient’s electronic medical records (n, %)	14 (14.6 %)	9 (14.1 %)	Metoprolol (extended-release) once a year or tacrolimus (extended-release) once daily 5 mg instead of 8 mg was registered in the patient’s electronic medical record
Other type of drug within same class in patient’s electronic medical records (n, %)	1 (1.0 %)	1 (1.6 %)	Atorvastatin was taken by a patient whereas pravastatin was registered in the patient’s electronic medical record

### Prevalence and Examples of Medication-Related Problems and Interventions Proposed for Medication-Related Problems

In total, 98 MRPs were identified in 53 patients, with a median of 2 (range 1–5) MRPs per patient. In 34 patients (53.1%) more than one MRP was identified during the consultation. Most frequent MRPs were: ADRs (22.4%), nonadherence (19.3%), unnecessary drugs (16.3%) and undertreatment of known comorbidities (12.2%).

In total, 113 interventions were proposed for the identified MRPs. In some cases, more interventions were proposed for one MRP. Interventions most frequently proposed were dosage optimization (21.2%), individualized recommendation regarding drug compliance (16.8%) and drug discontinuation (12.4%). Most interventions proposed by the CP (93.6%) were followed by both the patients and the hepatologists. Interventions proposed and not accepted by the hepatologist or the patient were interventions in which the hepatologist or the patient had to stop or change the time of administration of a drug that was started by the primary care physician. Interventions were not accepted due to uncertainties about the medication (e.g. indication or no causal relation with side-effects). One year after the consultation with the CP, 79% (15/19) of the patients experienced no compliance issues and 93% (27/29) of patients used no drugs with indication issues anymore. No patient experienced an unplanned hospital admission related to medication during the year after the consultation. Table 3 and 4 present the prevalence and some examples of MRPs and the interventions proposed for MRPs.

**TABLE 3:** Prevalence and examples of MRPs.

MRPs		*N* (%) instances of MRPs (*n* = 98)	Example of MRPs
Nonadherence	Intentional	7 (7.1 %)	Mycophenolic acid is taken once daily instead of twice daily
	Unintentional	12 (12.2 %)	Medication during day time or before bed is forgotten
ADR		22 (22.4 %)	Hypotension and dizziness by blood pressure lowering medication
Drug interaction	Drug - disease	3 (3.1 %)	Use of NSAIDs
Indication	Wrong drug	1 (1.0 %)	Xylometazolin nasal spray used for allergies
	Unneccessary drug	16 (16.3 %)	Use of PPI without indication
	Untreated indication	12 (12.2 %)	Patient with frequent migraine and untreated neuropathic pain
Suboptimal dose	Dose too high	2 (2.0 %)	Normal dose used despite kidney insufficiency
	Dose too low	2 (2.0 %)	Inadequate dose of PPI for prophylaxis of a gastro-intestinal bleeding
Monitoring issues		1 (1.0 %)	New drug started by other specialism which requires monitoring of the liver enzymes regularly
Experienced complexity in dosage regimen		5 (5.1 %)	Too many administration times for medicines e.g. 5 or 6 times daily
Logistic problems		5 (5.1 %)	Shortage of medicines in community pharmacy
Drug use problems		7 (7.1 %)	Problems with the taste of medicines
Other		3 (3.1 %)	Questions of patients about e.g. interactions, drug use and pregnancy and storage

ADR, Adverse Drug Reaction; e.g., for example; MRPs, Medication-Related problems; NSAID; nonsteroidal anti-inflammatory drug; PPI, proton pump inhibitor.

**TABLE 4:** Interventions proposed for MRPs.

Interventions proposed for MRPs		N (%) interventions proposed for MRPs (*n* = 113)	Examples of interventions
Optimizations	Dosage regimen	24 (21.2 %)	Simplification of complex medication schedules from 6 moments of intake to 3
	Stop	14 (12.4 %)	No indication for PPI or acetylsalicylic acid
	Start	8 (7.1 %)	Laxative for constipation due to opioid usage and sildenafil for erectile dysfunction
	Switch	1 (0.9 %)	Tacrolimus twice-daily formulation to once-daily formulation
Patient handling interventions	Education about medication	11 (9.7 %)	Explanation of the indication for xylometazoline nasal spray; not to be used to treat allergies and to be used for a maximum of 7 days
	Medication compliance advise	19 (16.8 %)	Information about how to organize medication intake properly, e.g. with the help of an application on your phone or an alarm
	Advise for practical problems with medicines use	8 (7.1 %)	Improving the intake of medication by giving advise how to mask the taste
	Advise on how to reduce ADRs	14 (12.4 %)	Changing the intake of blood pressure medication to the evening to prevent for dizziness
	Advise how to stop medication	2 (1.8 %)	Advise with regards to stop PPI use
	Refer to other health care professional	9 (8.0 %)	Patients with unclear indications for a medicines referred to specialist or general practitioner
			Patient experiences pain for a long time referred to pain consultant
Dispensing		3 (2.7 %)	Wrong tablets in multidose drug dispensing bags

ADRs, Adverse Drug Reactions; e.g., for example; MRPs, Medication-Related problems; PPI, proton pump inhibitor.

### Severity of the Medication-Related Problems

The majority of the MRPs (57.1%, 56/98) was rated in category D (error caused potential harm to patient). In total, 10 MRPs (10.2%) were rated in category E (error caused temporary harm and required intervention) and 1 (1%) MRP was rated in category F (error caused temporary harm and required hospitalization). MRPs rated in category E and F were: use of anticoagulants without indication, use of contra-indicated drugs, dose not adapted in patient with worse renal function, no use of prophylactic antibiotics with major dental surgery and wrong dose frequency of immunosuppressive agents. The other MRPs were rated in category A (22.4%, 22/98; events that have the capacity to cause error) and category C (9.2%, 9/98; .error occurred without posing harm to patients).

## Discussion

In this cohort, LTx recipients experience a median of 2 MRPs with the majority of the errors causing potential harm to patients (68.3%). ADRs, nonadherence and the use of unnecessary drugs were the most frequently reported MRPs in this cohort. Interventions most frequently proposed by the CP were dosage optimization, individualized recommendation regarding drug compliance and drug discontinuation. The clinical relevance of this program by the CP is shown by a reduction in patients experiencing compliance issues and patients using drugs with indication issues.

Our results are in line with Taber et al., who evaluated MRPs in kidney transplant recipients (Taber et al., 2012). They showed that MRPs commonly occur in kidney transplant recipients with nonadherence and ADRs as most frequently reported MRPs in their cohort. Recently, another study by Hayward et al. found results comparable to ours with nonadherence and indication issues as most frequently reported MRPs in a cohort of ambulatory patients with cirrhosis (Hayward et al., 2019). Interestingly, they found more drug interactions, dose issues and monitoring issues in comparison with our study. An explanation for this difference is the fact that in the Netherlands a nationwide electronical medication monitoring system is implemented with clinical decision support and clinical rules (van der Sijs et al., 2010; Beex-Oosterhuis et al., 2013). As a consequence, physicians and pharmacists receive drug safety alerts directly during prescribing preventing for suboptimal doses, drug interactions and monitoring issues (e.g. the measurement of through levels for certain drugs).

Most frequently proposed interventions were dosage optimization, individualized recommendation regarding drug compliance and drug discontinuation. Interestingly, most ADRs and nonadherence issues in this cohort were due to complex medication schedules. Furthermore, the use of unnecessary drugs was approximately one fifth of the MRPs, which shows that a comprehensive review of medication is not regularly performed by the treating physician during the outpatient visit.

MRPs and especially nonadherence have been found to be correlated to multiple factors such as socioeconomic, therapy-related and healthcare organizational (Belaiche et al., 2017). Methods used to improve nonadherence are automated prescription refill assistance, patient’s self-reports or eHealth applications. However, MRPs in LTx recipients can only be solved by multi-faceted interventions targeting behavioral, educational and emotional factors and providing multidisciplinary care including a consultation with a CP.

Interventions proposed by the CP were in 93.6% followed by both the patient and the hepatologist. Other international studies show comparable acceptance rates of approximately 95%, whereas studies in the Netherlands show an acceptance rate of approximately 80% (Kopp et al., 2007; Bosma et al., 2008). Probably, at the Erasmus University Medical Center an acceptance rate in accordance with international studies is achieved by a recent change in the workflow. CPs are dedicated to specific wards causing intensive collaborations with all health care providers. During some consultations the number of interventions initiated for the MRPs were greater than the total number of MRPs. This is caused by the fact that for some MRPs multiple interventions are possible. For example, nonadherence could be improved by medication optimizations and medication compliance advises. Also, ADRs could be solved by a change in the dosage regimen and an advice on how to reduce or handle adverse drug reactions. Moreover, potential medication related complications were prevented in 68.3% of the patients.

This newly established consultation with the CP is performed during the annual check-up. Possibly a consultation more frequently over the year might be more beneficial, even for recently transplanted patients. A potential hurdle for this kind of involvement of the pharmacist is current absence of financial reimbursement for the CP in the Netherlands. With a reduction in patients experiencing compliance issues and patients using drugs with indication issues we show the clinical relevance of this program. The results of this study implicate that an intensive collaboration between liver transplant healthcare professionals and pharmacists is needed and should be evolved in the near future.

Strengths of our study are the real-life clinical setting, the fact that the MRPs were independently categorized and the good collaboration between the Department of Hepatology and the Department of Hospital Pharmacy. Furthermore, we did assess the severity of the MRPs and evaluated the MRPs and proposed interventions one year after the consultation. As far as we know, this is the first study that describes MRPs in the outpatient setting focusing on liver transplant recipients. Our study has some limitations. Due to the fact that the consultation with the CP was planned one afternoon per week, not every LTx recipient monitored at the outpatient clinic of the Erasmus University Medical Center has been consulted by the CP. As a consequence, we might under- or overestimate the actual prevalence of MRPs in our cohort. However, patients of all hepatologists working at the Erasmus University Medical Center are seen by the CP. Therefore, we assume that this cohort is a good reflection of all LTx recipients monitored at the outpatient clinic. Furthermore, patient satisfaction about the consultation of the CP was not monitored. Further research could focus on this topic, together with the prevention of unplanned hospital admissions of LTx recipients. In the future, a randomized controlled trial could be performed evaluating the effect of a consultation provided by a CP that combines several strategies to reduce MRPs in LTx recipients. In summary, LTx recipients in this cohort experience a median of 2 MRPs of which ADRs, nonadherence and unnecessary drugs are most frequently reported. The clinical relevance of this program is shown by a reduction in patients experiencing compliance issues and patients using drugs with indication issues. An outpatient monitoring program of a CP for LTx recipients can signal MRPs and lead to interventions that are accepted by both the patients and the hepatologists and hence result in optimization of medication safety in LTx recipients.

## Data Availability

The raw data supporting the conclusions of this article will be made available by the authors, without undue reservation.
